# Pannexin 1 Is Critically Involved in Feedback from Horizontal Cells to Cones

**DOI:** 10.3389/fnmol.2017.00403

**Published:** 2017-12-07

**Authors:** Valentina Cenedese, Wim de Graaff, Tamás Csikós, Mitali Poovayya, Georg Zoidl, Maarten Kamermans

**Affiliations:** ^1^Retinal Signal Processing Lab, Netherlands Institute for Neuroscience, Amsterdam, Netherlands; ^2^Department of Biology, York University, Toronto, ON, Canada; ^3^Department of Biomedical Physics and Biomedical Optics, Academic Medical Center, University of Amsterdam, Amsterdam, Netherlands

**Keywords:** Pannexin 1, retina, horizontal cells, cones, feedback, connexins, lateral inhibition, zebrafish

## Abstract

Retinal horizontal cells (HCs) feed back negatively to cone photoreceptors and in that way generate the center/surround organization of bipolar cell receptive fields. The mechanism by which HCs inhibit photoreceptors is a matter of debate. General consensus exists that horizontal cell activity leads to the modulation of the cone Ca-current. This modulation has two components, one fast and the other slow. Several mechanisms for this modulation have been proposed: a fast ephaptic mechanism, and a slow pH mediated mechanism. Here we test the hypothesis that the slow negative feedback signal from HCs to cones is mediated by Panx1 channels expressed at the tips of the dendrites of horizontal cell. We generated zebrafish lacking Panx1 and found that the slow component of the feedback signal was strongly reduced in the mutants showing that Panx1 channels are a fundamental part of the negative feedback pathway from HCs to cones.

## Introduction

A fundamental feature of the vertebrate visual system is that many of its neurons have receptive fields with a center/surround organization. Such organization is crucial for phenomena like contrast enhancement and redundancy reduction (see for instance: Chalupa and Werner, 2003). The first place in the visual system, where the center/surround organization of receptive fields occurs, is in the outer retina, where cones, horizontal cells (HCs) and bipolar cells (BCs) interact at the cone synaptic terminal. Each HC receives input from multiple cones and neighboring HCs are strongly coupled electrically. Consequently, they have large receptive fields. HCs feed back negatively to cones and in that way generate the surround responses of the BCs via a process called lateral inhibition (see for instance: Chalupa and Werner, 2003). In addition to this there is also some evidence that HCs might feed back positively to cones (Jackman et al., 2011).

Despite the essential role in vision, played by this lateral inhibition process, its underlying mechanism has puzzled the neuroscience community for decades. The synaptic interaction between cones and HCs turns out to be surprisingly complex (see for reviews: Klaassen et al., 2012; Thoreson and Mangel, 2012; Kramer and Davenport, 2015; Chapot et al., 2017). General agreement exists that hyperpolarization of HCs leads to a shift of the activation function of the Ca-current (*I*_Ca_) in cones to more hyperpolarized potentials (Verweij et al., 1996). This has been found in all species studied so far (goldfish, zebrafish, mouse, newt, salamander, primate; Verweij et al., 1996, 2003; Hirasawa and Kaneko, 2003; Cadetti and Thoreson, 2006; Thoreson et al., 2008; Klaassen et al., 2011). However, there is less consensus regarding the mechanism that induces this shift. There is evidence that the modulation of *I*_Ca_ in cones is due to: (1) an ephaptic mechanism (Kamermans et al., 2001; Klaassen et al., 2012); (2) a pH dependent mechanism (Hirasawa and Kaneko, 2003; Davenport et al., 2008; Thoreson et al., 2008; Wang et al., 2014; Warren et al., 2016a); and (3) a GABAergic mechanism (Wu and Dowling, 1980; Tachibana and Kaneko, 1984; Tatsukawa et al., 2005; Hirano et al., 2016).

The ephaptic mechanism strongly depends on connexin (Cx) hemichannels. Cxs are proteins that form gap-junction; i.e., large ion-channels between cells. In some occasions they can function as hemichannels (Kamermans et al., 2001). These hemichannels are non-selective, highly conductive, transmembrane ion channels. Current flows into the HC dendrites via the Cx-hemichannels, which generates a slight negativity deep in the synaptic cleft of the cone synaptic terminal. This negativity is sensed by the Ca-channels of the cone and is visible as a shift of the activation function of *I*_Ca_ to negative potentials (Kamermans et al., 2001). When Cx-hemichannels at the tips of HCs dendrites are lacking, feedback responses in cones are reduced (Shields et al., 2007; Klaassen et al., 2011). The ephaptic feedback pathway, being purely electrical, is extremely fast (synaptic delay <1 ms; Vroman et al., 2014).

There is no consensus about the pH mechanism. When discussing the mechanism by which the pH in the synaptic cleft is modulated, it is good to consider the properties of such a mechanism. As argued in Vroman et al. (2014), it is unlikely that protons are taken up or released, since this would be highly unreliable because of the very small amount of free protons available in the synaptic cleft. A change in pH buffer capacity is needed for a reliable change in pH. Vroman et al. (2014) suggested that pannexins were involved in this pH mechanism. Pannexins (Panx) are proteins related to Cxs. However, Panxs do not form gap-junctions but function as transmembrane channels. Just as Cx-hemichannels, these Panxs-channels are non-selective, highly conductive, ion channels. Furthermore, Pannexin1 (Panx1) is implicated in ATP release (Bao et al., 2004; MacVicar and Thompson, 2010). Vroman et al. (2014) proposed that the pH dependent mechanism relies on ATP released via Panx1 channels that are expressed at the tips of HC dendrites. Nucleoside triphosphate diphosphohydrolase-1 (NTPDase1) and Adenosine deaminase (ADA) hydrolyzes the ATP released in the synaptic cleft into inosine, phosphate groups, and protons. This generates a phosphate buffer that acidifies the synaptic cleft and inhibits *I*_Ca_ in cones. Hyperpolarization of HCs leads to the closure of the Panx1 channels thereby reducing ATP release. This in turn leads to a reduction of the phosphate buffer concentration in the synaptic cleft, which then alkalizes, and *I*_Ca_ increases. This Panx1/ATP pathway is very slow (time constant is ≥200 ms; Vroman et al., 2014).

Warren et al. (2016a) proposed that HCs regulate the proton concentration in the synaptic cleft via modulation of the Na^+^/H^+^ exchanger. The main problem with this suggestion is that this transporter is electrically neutral and therefore cannot modulate the proton concentration in a HC membrane potential dependent manner; a prerequisite for being the feedback mechanism. The authors acknowledged this and suggested that the release of bicarbonate might also be involved. Could it be that a bicarbonate buffer is being modulated in the synaptic cleft instead of the phosphate buffer? The fundamental difference between the two is that a bicarbonate buffer is a so-called “open buffer” whereas the phosphate buffer is a “closed buffer”. In a closed buffer system the sum of the concentrations of the bound and unbound buffer component remains equal, whereas in an open buffer system this can change depending on the pH. CO_2_ can be formed and escape the system easily by diffusion. This difference is essential. The bicarbonate concentration is in equilibrium with the local CO_2_ concentration. The conversion of H_2_O and CO_2_ into bicarbonate is catalyzed by the enzyme carbonic anhydrase, which is one of the fastest regulatory enzymes known (Stryer, 1981). This enzyme is present in the synaptic cleft (Fahrenfort et al., 2009), which means that the amount of bicarbonate will strongly depend on the local CO_2_ concentration. This will be controlled by all the cells near the synapse, like cones, HCs, BCs and Müller cells. As such, the system is an unreliable way of modifying the pH buffer capacity in a manner that only depends on the HC membrane potential.

The third proposed feedback mechanism is GABA. Endeman et al. (2012) showed that GABA inhibits negative feedback on a very slow timescale. It is a neuromodulator. Kemmler et al. (2014) came to a similar conclusion for mouse retina. Additionally, Liu et al. (2013) showed that GABA-receptors are permeable for HCO_3_^−^. So activating GABA receptors might, in addition to the effect it will have on the HC membrane potential, also affect the basal pH in the synaptic cleft to some extent. Both these actions will indirectly affect feedback. This might be the reason that mice lacking the vesicular GABA transporter in HCs (Cx57-VGAT^−/−^) have disturbed feedback responses (Hirano et al., 2016).

Vroman et al. (2014) showed that in goldfish and zebrafish the ephaptic- and pH-feedback mechanisms are both present and generate a feedback system that has a fast and a slow component. Warren et al. (2016b) confirmed that there are two components to feedback with different kinetic properties. In the present study we set out to test the involvement of Panx1 channels in feedback from HCs to cones using genetic methods, because pharmacological manipulations that interfere with the extracellular pH, often induce additional confounding effects (see for instance: Fahrenfort et al., 2009).

Zebrafish express two isoforms of Panx1—Panx1a and Panx1b. In order to determine the role of either Panx1, we generated zebrafish (*Danio Rerio*) lacking these isoforms using transcription activator-like effector nuclease (TALEN) technology. The lack of Panx1a or Panx1b or both did not affect the development or organization of the outer retina. However, feedback responses measured in cones were strongly reduced, especially the slow component of feedback. Previously we have shown that the fast component of feedback in Cx55.5^−/−^ animals is reduced (Klaassen et al., 2011). Thus, feedback responses in zebrafish with mutations in the two key proteins of the negative feedback pathway (Cx55.5—fast feedback and Panx1—slow feedback) were strongly reduced in a manner consistent with the model proposed by Vroman et al. (2014).

## Materials and Methods

### Experimental Animals

This study was carried out in accordance with the recommendations and the approval of the Competent Authority (CCD-license AVD801002017830), the ethical committee of the Royal Netherlands Academy of Arts and Sciences (DEC-KNAW) and the Animal Welfare Body of the Netherlands Institute for Neuroscience (IvD-NIN), acting in accordance with the European Communities Council Directive 2010/63/EU.

Wild type zebrafish, *Danio Rerio*, (TL strain) were obtained from the Zebrafish International Resource Center (Eugene, OR, USA; NIH-NCRR grant #RR12546). Male and female fish were maintained in aquaria at 28° to 28.5°C under a 14/10 h light/dark cycle. Zebrafish were fed 2–3 times a day on a diet of live *Artemia nauplii* (INVE Technologies, Dendermond, Belgium; INVE Aquaculture, Salt Lake City, UT, USA) and dry food (Luckstar feed #0.2; Catvis, Netherlands). Zebrafish lines were propagated by natural mating initiated by the onset of light. Embryos were grown at 28° to 28.5°C in E3 containing 1 × 10^−5^% methylene blue (Sigma, St. Louis, MO, USA) under standard conditions. In the present study, wild-type and Panx1a^−/−^, Panx1b^−/−^, Panx1a^−/−^/Panx1b^−/−^, Cx55.5^−/−^ (ZFIN ID: ZDB-ALT-1109020-1) and Cx55.5^−/−^/Panx1a^−/−^ mutant zebrafish were used.

### Generation of the Mutant Zebrafish

**Figure 1:**
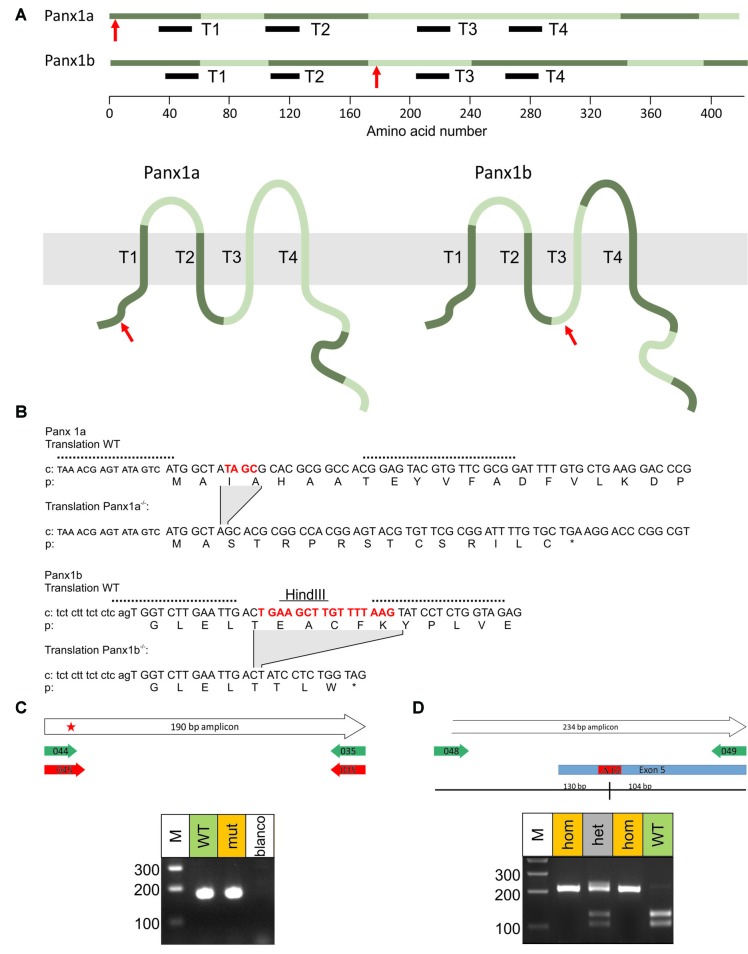
Protein map Panx1 (Ensembl). **(A)** Schematic representation of the two Panx1 genes in Zebrafish. Panxs are proteins with four transmembrane domains, two highly conserved extracellular loops, one intracellular loop and a long C-terminal end. Panx1a has six exons. The first exon, which contains the coding sequence for a transmembrane domain, was targeted. The mutation site that leads to an early stop-codon is indicated with the red arrow. Panx1b has seven exons. The fourth exon, which contains the coding sequence for the third transmembrane domain was targeted. The site of the mutation is indicated by the red arrow. Alternating exons are indicated in light and dark green. **(B)** Part of the cDNA (c) and protein (p) sequence flanking the mutated site for Panx1a and Panx1b. In upper case letters, the coding sequence/amino acids, in small capitals the 5′-UTR and in lower case letters the intron region. In red the deleted base pairs. The asterisk indicates the premature stop of the protein. The dotted lines indicate the target regions of the TALENS. The solid line indicates the restriction site HindIII. **(C)** Panx1a mutation identification. PCR results of the amplicons of the WT primer pair in WT and the amplicons of the mutant primer pair in mutant. Both amplicons have the expected size. **(D)** Panx1b mutation identification. The PCR amplicons of WT would be cut with HindIII resulting in two smaller bands. In the mutant the restriction site is absent and HindIII was unable to cut the amplicon. This resulted in only one band. As expected, in the heterozygote animals three bands occur.

The Cx55.5^−/−^ mutant zebrafish were generated using the TILING technique and have been described before (Klaassen et al., 2011). Panx1^−/−^ mutant zebrafish were generated using TALENS technology described by Bedell et al. (2012) and Sanjana et al. (2012). TALENs recognizing their DNA-binding sites via protein domains, were modularly assembled for each DNA target using the GoldyTALEN modified scaffold (Bedell et al., 2012). TALEN target sequences were selected for Panx1a (Gene ID: 393890): left target sequence—TAAACGAGTATAGTCATG, right target sequence—GGAGTACGTGTTCGCGGA (exon 1) and for Panx1b Gene ID: 567417: left target sequence—CAGTGGTCTTGAATTG, right target sequence, AAGTATCCTCTGGTAG (exon 4; dotted lines in Figure 1B). *In vitro* transcribed mRNA (mMESSAGE mMACHINE^®^ Ultra Kit, Ambion) encoding each TALEN pair was injected into one-cell stage ZF-embryos. Amounts of 25–400 pg (1 nl) RNA were used. PCR primers used to screen for mutants were for Panx1a FWD_TGTCCTCGCTTGAGTATTCGC, REV_ AGAAACTTCCTGAGCGAAGGC and for Panx1b FWD_ATTCTACCATTCTTACATGAATTCCCT, REV_ GAGGAAGGTTAAAACCCGACAAAGC. Founders were outcrossed one or several times and then incrossed for maximal two generations to generate homozygous mutants. Only single or double homozygous mutants and their closely related wild-type littermates were analyzed in this study.

### Immunohistochemistry

Immunohistochemical procedures were similar to published methods (Klaassen et al., 2011; Vroman et al., 2014). Various antibodies labeling key features of the outer retina were used. Cx55.5 is present at in the gap-junctions between HCs and at the tips of the HC dendrites. In this article we will focus on the gap-junctions. The primary antibody against Cx55.5 (1:5000) was raised in rabbit and described in Shields et al. (2007). Mouse monoclonal antibodies against the ionotropic glutamate receptor GluR2 (1:200) and GluR4 (1:100) were obtained from Chemicon (Temecula, CA, USA), and Fret43 (1:200) a marker for double cone synaptic terminals in zebrafish (Larison and Bremiller, 1990), was a kind gift from Ms. R. Bremiller (University of Oregon, Eugene, OR, USA). The primary antibody against NTPDase1 (1:500) was raised in rabbit to the corresponding amino acid sequence 102–130 of human NTPDase1 (GIYLTDCMERAREVIPRSQHQETPVYLGA) and was obtained from CHUQ^1^ and characterized by Ricatti et al. (2009). Secondary antibodies, goat-anti-mouse Cy3 and goat-anti-rabbit Cy3, were purchased from Jackson Immuno Research Lab (West Grove, PA, USA).

Cryosections (10 μm) were made and stored at −20°C. Sections were washed with PBS and pre-incubated in Blocking Buffer (1× PBS/5% normal goat serum/0.3% Triton™ X-100), then incubated with primary antibodies (dilutions in 1× PBS/1% BSA/0.3% Triton™ X-100) for 18 h followed by three PBS washes. After that, they were incubated with secondary antibodies (2 h room temperature), washed three times with PBS and mounted in Vectashield with DAPI. 0.25 μm optically sectioning was performed with Leica SP5 confocal microscope (Leica, Germany).

### Retina Preparation

Zebrafish were dark-adapted in a light-tight chamber for 15 min, euthanized by immersion in iced water, and decapitated. The head was bisected along the anterior/posterior axis, the eyes were removed and placed in oxygenated Ringer solution. An eyecup was prepared under red light illumination by removing the cornea, lens, and as much vitreous humor as possible. Each retina was removed from the eyecup with fine forceps. Isolated retinas were mounted (photoreceptors up) to a small piece of tissue paper and placed in a recording chamber. The ends of the tissue paper were held securely in place under a harp. Retinas mounted in this manner were continuously superfused with Ringer’s solution (1 ml/min). All steps of the procedure were carried out in the presence of dim red light.

### Electrophysiology

#### Electrodes and Recording Equipment–Voltage Clamp Recordings

Whole-cell voltage clamp recordings were made from cones. The recording chamber containing the isolated retina was mounted on a Nikon Eclipse E2000FN microscope (Nikon, Japan) and viewed with a Nikon 60× water immersion objective with infrared differential interference contrast and a video camera (Philips, Netherlands). Recording electrodes were pulled from borosilicate glass (BF-150-110-10; Sutter Instruments, Novato, CA, USA) with a Flaming/Brown micropipette puller (Model P-1000; Sutter Instruments, Novato, CA, USA). The impedances ranged from 8 MΩ to 12 MΩ when filled with pipette medium and measured in bathing solution. Pipettes were connected to an Axopatch 200A patch clamp amplifier (Molecular Devices, Sunnyvale, CA; four-pole low-pass Bessel filter setting, 2 kHz). Signal software (v. 3.07; Cambridge Electronic Design (CED), Cambridge, UK) was used to generate voltage command outputs and to acquire data. Signal software (v. 3.07; CED), MatLab (v2016b, MathWorks), Igor.pro (WaveMetrics, Portland, OR, USA) and Origin Pro (v8, Origin Lab Corporation), were used to analyze the data. All data shown are corrected for the junction potential.

#### Electrodes and Recording Equipment–Intracellular Recordings

HC responses were recorded with intracellular recording techniques. Microelectrodes were pulled on a horizontal puller (Sutter P-80-PC; San Rafael, CA, USA) using aluminosilicate glass (OD = 1.0 mm, ID = 0.5 mm; Clark, UK) and had impedances ranging from 100 MΩ to 300 MΩ when filled with 3 M KCl. The intracellular recordings were made with a WPI S7000A microelectrode amplifier system (World Precision Instruments, USA), and sampled using an AD/DA converter (CED 1401, Cambridge Electronic Design, UK) coupled to a Windows based computer system.

#### Optical Stimulator

A 20 μm white light spot was focused via a 60× water immersion objective on the cone outer segment and a 4500 μm “full field” stimulus could be projected through the microscope condenser. The light stimulator consisted of two homemade LED stimulators based on a three-wavelength high-intensity LED (Atlas, Lamina Ceramics Inc., Westhampton, NJ, USA). The peak wavelengths of the LEDs were 624, 525 and 465 nm, respectively, with bandwidths smaller than 25 nm. An optical feedback loop ensured linearity. The output of the LEDs was coupled to the microscope via light guides. White light consisted of an equal quantal output of the three LEDs. Zero log intensity was 8.5 × 10^15^ quanta m^−2^s^−1^.

#### Electrode and Bath Solutions

The standard patch pipette medium contained (in mM): 21 KCl, 85K-gluconate, 1 MgCl_2_, 0.1 CaCl_2_, 1 EGTA, 10 HEPES, 10 ATP-K_2_, 1 GTP-Na_3_, 20 phosphocreatine-Na_2_ and 50 units/ml creatine phosphokinase. The pH of the pipette medium was adjusted to 7.2 with KOH and resulting in an E_Cl_ of −50 mV when used in conjunction with the Ringer solution.

The bath solution contained in (mM): 102 NaCl, 2.6 KCl, 1 CaCl_2_, 1 MgCl_2_, 5 D-glucose, and 28 NaHCO_3_, and was continuously gassed with 2.5% CO_2_ and 97.5% O_2_ to yield a pH of 7.6. All chemicals were obtained from Sigma-Aldrich (St. Louis, MO, USA). Bath solution flowed continuously through the recording chamber via a gravity-driven superfusion system. Switching between bathing solutions was achieved through computer-controlled valves. During all electrophysiological experiments with the isolated retina, GABAergic transmission in the retina was blocked by 200 μM Picrotoxin (PTX).

#### Measuring Light-Induced Feedback in Cones

Cones were clamped at −60 mV. The cone type was determined with 500 ms light flashes of different wavelengths (465 nm, 525 nm and 624 nm). After this classification, the cone was saturated with a spot of light (20 μm diameter) of the optimal wavelength for that cone. Subsequently, a full-field stimulus of white light (500 ms) was given while the cone was voltage clamped at potentials ranging from −80 to +20 mV. The feedback responses were measured at the voltage that yielded the biggest responses (around −50 mV).

#### Method for Measuring the Time Constants of Feedback

Following Vroman et al. (2014), the feedback responses were fitted with a double exponential function (Eq. 1):

(1) Y=Af(1−e−tτf)+As(1−e−tτs)

where *A*_f_ and *A*_s_ are the amplitudes of the fast and slow feedback component respectively and *τ*_f_ and *τ*_s_ are the time constants of the fast and slow feedback component, respectively.

Because of the small size of the feedback responses in the various zebrafish mutants, we were unable to fit the double exponential function adequately when *A*_f_, *A*_s_, *τ*_f_ and *τ*_s_ all were free parameters. To estimate the contribution of both feedback components, we assumed that the time constants of the two feedback components (*τ*_f_ and *τ*_s_), were independent of the genotype and used the estimates of the time constants from Vroman et al. (2014): *τ*_f_ = 300 ms and *τ*_s_ = 300 ms. That left only the amplitudes of the two feedback components (*A*_f_ and *A*_s_) as free parameter. The fitted amplitudes were used as an estimate for the relative contribution of the feedback pathways in the various mutants.

#### Method for Measuring the Half Activation Potential of *I*_Ca_

*I*_Ca_ was isolated by leak subtracting the whole cell IV-relation. The leak was estimated in the linear part of the IV relation between −80 mV and −60 mV (Vroman et al., 2014) and subtracted from the whole cell IV-relation. The resulting IV-relation of *I*_Ca_ was normalized relative to its negative peak and the half activation potential of the normalized *I*_Ca_ was estimated by fitting a Boltzmann relation (Eq. 2) in the voltage range from −60 mV to the voltage generating the peak Ca-current.

(2) A=11+e−(V− K)n

where *A* is the activation of *I*_Ca_, *V* is the holding potential, *K* is half activation of *I*_Ca_, and *n* is the slope factor.

### Statistical Analysis

Data are presented ± the standard error of the mean (SEM). Levels of significance were determined using Student’s *t*-test or a Pearson’s *χ*^2^ test. Data were considered significantly different for *p*-values smaller than 0.05. Curve fitting and statistical analyses were performed using SPSS, or Igor.pro software.

## Results

### Generation of the Mutant Zebrafish

To eliminate Panx1 function we generated knock out zebrafish using the TALENS technique (see “Materials and Methods” section). For Panx1a we targeted a site early in exon 1 (Figure 1) and selected a mutant c.8delta GATC, a 4 bp deletion leading to a scrambled protein (downstream of the second amino acid) and an early stop-codon at amino acid 16. A specific PCR was designed for genotyping this mutant: FWD_CGAGTATAGTCATGGCTATA for the WT allele, FWD_GAGTATAGTCATGGCTAGCA for the mutant allele and REV_AGAAACTTCCTGAGCGAAGGC for both (Figure 1C).

For Panx1b, we targeted a site early in exon 4, which has been shown to lead to a loss of Panx1 function in mouse (Dvoriantchikova et al., 2012; Prochnow et al., 2012; Figure 1). Exon 4 codes for the intracellular loop and the third transmembrane domain. We selected a mutant c.534deltaTGAAGCTTGTTTTAAG, a 16 bp deletion leading to an early stop-codon three amino acids downstream of the deletion. Mutants could be identified by PCR, using the primers described (see “Materials and Methods” section) and digest the resulting 234 bp amplicon with HindIII. WT alleles were cut into 130 bp and 104 bp, whereas mutant alleles were not (Figure 1D). The selected mutations lead to truncated proteins that will be incapable of generating functional channels (see also “Discussion” section). Proof at the protein level that the Panx1a or the Panx1b protein was not present in the mutants was not possible because of the lack of specific antibodies. Both the single and double homozygous mutant zebrafish developed normally.

### Morphological Analysis of Mutant Zebrafish

**Figure 2:**
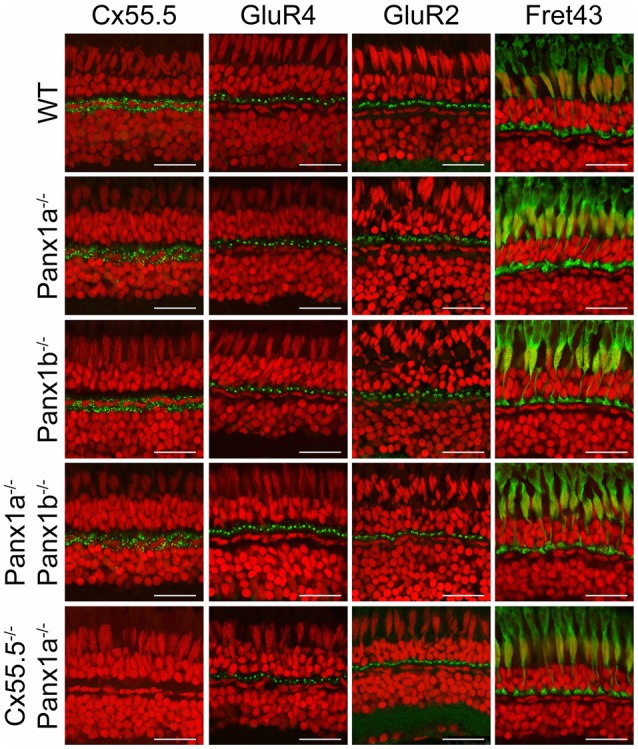
Overview of marker proteins for outer retinal function. Immunocytochemical staining for Cx55.5 (horizontal cells (HCs) gap-junctions), GluR4 (glutamate receptors on OFF-BC dendrites), GluR2 (glutamate receptors on HC dendrites) and Fret43 (synaptic terminals of double cones). Expression of these proteins did not differ between WT and the various mutant animals (Pax1a^−/−^, Panx1b^−/−^, Panx1a^−/−^/Panx1b^−/−^, Cx55.5^−/−^/Panx1a^−/−^). Scale bar is 25 μm.

Next, we determined whether the overall organization of the outer retina was intact in the various mutant zebrafish. Figure 2 shows the results of immunocytochemical analysis of the outer retina of the various zebrafish lines. Cell bodies are labeled by DAPI and marked in red while the cell-specific markers are shown in green. The following cell-specific markers were used: Cx55.5: gap-junctions between HCs; GluR4: glutamate receptor on OFF-BC dendrites; GluR2: glutamate receptor on HC dendrites; FRet43: a marker for double cones synaptic terminals.

Cx55.5-immunoreactivity (IR) is visible as puncta around the HCs somata (Figure 2, first column). The Cx55.5-IR in the various Panx1^−/−^ animals did not differ from that of the WT but was absent in the Cx55.5^−/−^/Panx1a^−/−^ animals. GluR4-IR (Figure 2, second column) is visible as green puncta at the level of the cone synaptic terminal, where BCs make contact with photoreceptors and did not differ between the WT and the various mutant animals. GluR2-IR is found in typical horse-shoe shapes, reflecting the HCs dendrites in the invagination of the cone synaptic terminal (Figure 2, third column). Both the localization and the shape are maintained in mutant animals. Fret43-IR (Figure 2, fourth column) is diffusely present in the whole synaptic terminal and is indistinguishable between the WT and mutants.

**Figure 3:**
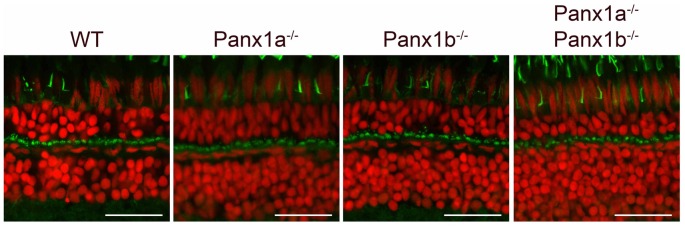
Expression of NTPDase1 in the outer retina does not differ between WT and the various genotypes (Pax1a^−/−^, Panx1b^−/−^, Panx1a^−/−^/Panx1b^−/−^, Cx55.5^−/−^/Panx1a^−/−^). Scale bar is: 25 μm.

NTPDase1 is the enzyme involved in the hydrolysis of ATP in the synaptic cleft (Vroman et al., 2014) and hence a critical component of the Panx1/ATP mechanism. NTPDase1-IR (Figure 3) is visible as bar-like blobs at the location of the cone synaptic terminal in WT and all the mutants indicating that the overall structure of the outer retinal is intact in the various mutants.

### Basic Properties of Cones and HCs in Panx1 Mutants

Next, we determined whether the mutations changed the resting membrane potential (*V*_rest_) of cones. The results in Table 1 indicate that there no significant changes in these parameters.

**Table 1:** Cone resting membrane potentials.

*V*_Rest_	Mean (mV)	SEM	*n*	*p*
WT	−43.67	1.48	6	
Pan1a^−/−^	−46.40	1.36	5	0.207
Panx1b^−/−^	−48.20	1.98	5	0.105
Panx1a^−/−^/Panx1b^−/−^	−37.00	6.34	5	0.358
Cx55.5^−/−^/Panx1a^−/−^	−46.25	4.40	4	0.610

*p values for WT vs. mutant tests*.

### Feedback Amplitude Is Reduced in Mutants

**Figure 4:**
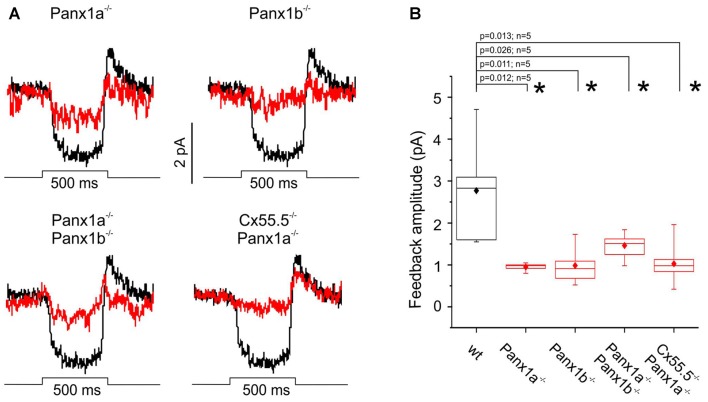
Light induced feedback responses in cones. Cones were saturated with a small spot of light and voltage clamped at −50 mV. A full field white light stimulus was applied. This resulted a small inward current: the light induced feedback response. **(A)** The black traces represent the mean light induced feedback response in WT. The red traces show the mean response of the various mutants. When Panx1a or Panx1b was mutated, feedback responses reduce but seem to keep a fast light-onset response. However, in the Cx55.5^−/−^/Panx1a^−/−^ animals the response on-set seems to be slowed down. **(B)** Quantification of the sustained feedback response for all the mutants. All mutants showed a significantly reduced feedback response. *p < 0.05.

Panx1 has been suggested to be involved in negative feedback from HCs to cones (Vroman et al., 2014). Therefore, we measured the negative feedback induced responses in cones. Cones in the isolated zebrafish retina were voltage clamped at E_Cl_ (−50 mV) and their direct light response was saturated by a bright small spot of 525 nm light. The retina was stimulated for 500 ms with a full field flash of white light to hyperpolarize HCs. Hyperpolarization of HCs leads to a shift of the activation potential of *I*_Ca_ of the cones to more negative potentials, which is visible as an inward current. Figure 4A shows the mean feedback induced response in WT (black traces). The maximal feedback response amplitude in WT was 2.76 ± 1.17 pA (*n* = 6). In all mutants, the feedback responses were significantly reduced (Figure 4A). Statistics are given in Figure 4B.

The feedback responses measured in cones could be reduced by a reduction of the feedback pathway from HCs to cones or indirectly by reducing the horizontal cell responses. Therefore we measured the responses of HCs to full field green light stimuli and found that the response amplitudes did not differ between WT and the various mutants (Table 2).

**Table 2:** Horizontal cell response amplitude.

Response amplitude	Mean (mV)	SEM	*n*	*p*
WT	−1.44	0.28	14	
Pan1a^−/−^	−1.24	0.16	12	0.559
Panx1b^−/−^	−1.01	0.13	8	0.280
Panx1a^−/−^/Panx1b^−/−^	−2.00	0.86	5	0.425

**Figure 5:**
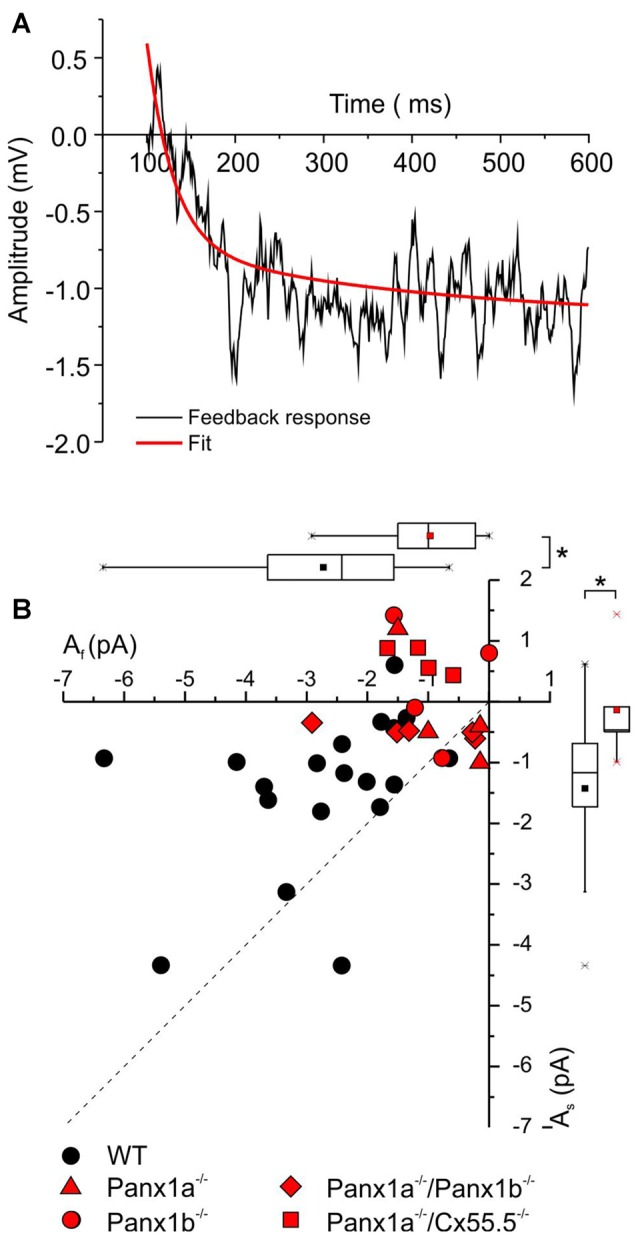
Quantification of the two feedback components. A double exponential function was fitted through the feedback responses (See “Materials and Methods” section). **(A)** Representative feedback trace (black) with fitted curve (red). **(B)** Scatter plot of the fast (x-axis) and the slow (y-axis) feedback component for WT (black symbols) and the Panx1^−/−^ animals combined (red symbols). The dotted line indicates equal amplitude for the fast and the slow feedback component. All the WT points are above this line, indicating that the amplitude of the fast feedback component is the largest. In the Panx1^−/−^ animals both the fast and the slow feedback component are significantly reduced. *p < 0.05.

Previously we have shown that feedback responses are best described by a double exponential function and suggested that Panx1 is involved in the slower component of the feedback pathway from HCs to cones. The prediction is that in the Panx1^−/−^ animals the slow component of feedback is more reduced than the fast component. Since the response amplitude of the feedback responses in zebrafish was rather small, we had to adapt the following strategy to fit the two exponential functions. We assumed the mutations would only affect the amplitudes (*A*_f_ and *A*_s_, respectively), but not the time constants, of the two feedback processes. Under this assumption we fitted a double exponential function through the feedback responses using the time constants determined by Vroman et al. (2014; Figure 5A). Figure 5B shows a scatter plot of the amplitudes of both components for the WT (black symbols) and the Panx1^−/−^ animals (red symbols). The dotted line indicates equal amplitude of both feedback components. The majority of the WT points are above the dotted line, indicating that the fast component is larger in WT than the slow component (A_f_: −2.72 ± 1.44 pA, *n* = 19; A_s_: −1.43 ± 1.27, *n* = 19; *p* = 0.006). In the Panx1^−/−^ the amplitude of the both the fast and the slow component were significantly reduced (A_f_-WT: −2.72 ± 1.44 pA, *n* = 19; A_s_-Panx1^−/−^: −0.97 ± 0.82 pA, *n* = 13; *p* = 0.0004 and A_s_-WT: −1.43 ± 0.29 pA, *n* = 19; A_s_-Panx1^−/−^: −0.15 ± 0.22 pA, *n* = 13; *p* = 0.003). A_s_ did not differ from zero in the Panx1^−/−^ animals (*p* = 0.5). In contrast, in WT A_s_ differs significantly from zero (*p* = 0.0001) and consist of about 43.6 ± 11.4% of the total feedback amplitude. This indicates that Panx1 is mostly involved in the slow feedback components but that it contributes to the fast feedback component as well.

### Relationship Is Shifted in Mutants

So far, we have shown that all Panx1^−/−^ mutants have reduced light induced feedback responses. However, in the dark, at the resting membrane potential of the cones and HCs, the feedback pathway is active as well. Therefore, we asked how the activation functions of *I*_Ca_ in cones in these animals was affected in an unstimulated condition. Whole cell IV-relations were obtained by measuring cones currents in response to a series of voltage steps while in the dark and then isolating *I*_Ca_ by leak-subtraction. A Boltzmann curve was fitted through the IV relation of *I*_Ca_ (see “Materials and Methods” section) and the half maximal activation values (*K*_half_) were determined.

**Figure 6:**
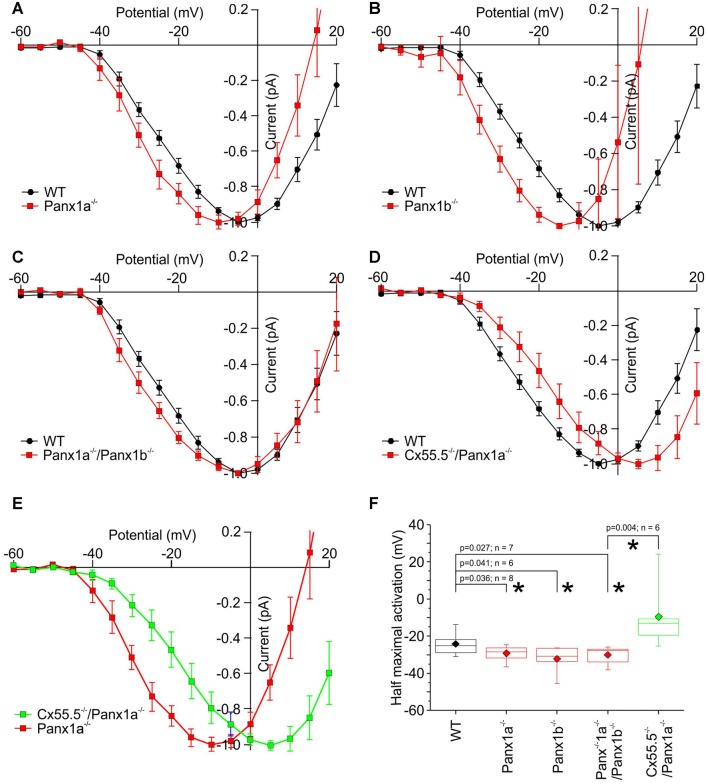
Normalized IV-relations of the cone Ca-current in WT and the various mutant animals. **(A–C)** In all Panx1 mutants the half activation potential of the Ca-current shifts to negative potentials. **(D)** In the Cx55.5^−/−^/Panx1a^−/−^ animals, the Ca-current shift slightly to positive potentials. **(E)** The activation function of *I*_Ca_ has shifted strongly to positive potentials in the Cx55.5^−/−^/Panx1a^−/−^ animals compared to the Panx1a^−/−^ animals (Red curve). **(F)** Quantification of the shifts of the Ca-current in cones. *p < 0.05.

Figures 6A–C show that the activation function of *I*_Ca_ is shifted to negative potentials for both Panx1^−/−^ animals and the double Panx1a^−/−^/Panx1b^−/−^ animals. For the Cx55.5^−/−^/Panx1a^−/−^ animals *I*_Ca_ showed a small shift to positive potentials relative to WT (Figure 6D) and a significant positive shift relative to the Panx1a^−/−^ animals (Figure 6E). Figure 6F gives the statistics of the half activation potentials. This is exactly what one would expect. Removing Panx1 from the HC dendrites leads to a reduction of the pH buffer concentration which alkalizes the synaptic cleft thereby relieving the inhibition on *I*_Ca_ (Vroman et al., 2014). The consequence is that the activation function of *I*_Ca_ shifts to negative potentials. Removing Cx-hemichannels will lead to an opposite shift. We have previously shown that blocking or removing Cx-hemichannels from the HC dendrites leads to a dissipation of the ephaptically induced negativity in the synaptic cleft and a shift of the activation function of *I*_Ca_ to positive potentials (Kamermans et al., 2001; Klaassen et al., 2011). This is consistent with the observation that the activation function of *I*_Ca_ in Cx55.5^−/−^/Panx1a^−/−^ has shifted to positive potentials relative to the Panx1a^−/−^.

## Discussion

In the present article we show that Panx1 channels are critically involved in negative feedback from HCs to cones. Knocking out Panx1 channels leads to a strong reduction of negative feedback. The main reduction of the feedback response seems to be due to a reduction of the slow feedback component. Previously it was shown that knocking out Cx55.5 led to a reduction in the fast feedback component (Klaassen et al., 2011). The results of this study are thus consistent with the feedback pathways as proposed by Vroman et al. (2014): a fast ephaptic feedback pathway mediated by Cx55.5 and a slow feedback pathway mediated by Panx1 channels.

We have not been able to generate a specific antibody for Panx1a and Panx1b. Therefore, we cannot show directly that Panx1a and Panx1b proteins are absent in the mutant fish. Is there a chance, given the mutation in both coding sequences that the gene-products of these mutated genes would lead to functional channels? The mutations in both the Panx1a^−/−^ and the Panx1b^−/−^ fish leads to an early stop codon. The resulting gene-products do not contain all four transmembrane domains and thus cannot form functional channels. Could exon skipping lead to functional proteins? Again in either case this is highly unlikely as a whole transmembrane domain is incorporated in the targeted exon. If exon skipping would have occurred, a protein with three transmembrane domains will be generated and such a protein will not form a functional channel. Furthermore, for Panx1b the same site has been targeted in mice and resulted in the loss of both Panx1 protein and function (Dvoriantchikova et al., 2012; Prochnow et al., 2012).

### Cx55.5 and Panx1 Are Both Involved in Feedback

The development of the retina of the Panx1 mutants was similar to WT. No changes in the distribution of key synaptic proteins, nor changes in the basic properties of cones were found in the mutants. However, feedback responses were substantially reduced while the HC response amplitudes were unaffected in all mutant animals. Detailed analysis of the shape of the feedback responses revealed that the slow feedback component was affected mostly by the loss of Panx1 channels. The fast feedback component was reduced as well. Does this mean that the Panx1 are involved in both the slow and the fast feedback component? Panx1 channels are large pores in the membrane and are implicated in the release of ATP and will also conduct an inward current. The size of this current will strongly depend on the membrane potential of the HC. Since the Panx1 channels and the Cx55.5 hemichannels are both located at the tips of the HC dendrites, they will contribute to the ephaptic feedback mechanism as well.

Is there evidence that Panx1a and Panx1b have different functions? Kurtenbach et al. (2013) showed that Panx1a and Panx1b have slightly different single channel properties. However, the present study does not show a strong difference between the behavior of the Panx1a^−/−^ and Panx1b^−/−^ animals. Knocking out either one of them leads to a strong reduction in feedback. The reason for the lack of a difference between the Panx1a^−/−^ and Panx1b^−/−^ animals might be that the Panx1 channels are strongly involved in the slow feedback pathway and that subtle changes in channel kinetics are not visible in the feedback measurements reported in this article. Interestingly the effects of Panx1a and 1b on feedback are not simply additive. Relative to WTs the feedback responses of the Panx1a^−/−^/Panx1b^−/−^ mutants were reduced by about the same amount as those of either the Panx1a^−/−^ or the Panx1b^−/−^ mutants. This might indicate that Panx1a and Panx1b form channels together and that the presence of both is required for properly functioning channels.

Is there evidence that Cx55.5 is involved in ATP release? In the double Panx1a^−/−^/Panx1b^−/−^ mutant the slow feedback component does not seem to be blocked completely. However, given the small feedback responses left in the Panx1^−/−^ mutants, it is unlikely that the Cx55.5 hemichannels contribute strongly to the ATP pathway. So, in summary, it seems that Cx55.5-hemichannels, Panx1-channels and glutamate receptors (see: Fahrenfort et al., 2005) mediate the ephaptic feedback component while only the Panx1 channels seem to mediate the ATP feedback pathway.

Both the Cxs and the Panxs mutants will be instrumental to further characterize the feedback pathways from HCs to cones. Combining these mutants with pharmacology might be an obvious thing to do but the interpretation of the will also suffer from the limited specificity of some of this available pharmacological tools used. For instance the application of high dose of HEPES, which is generally assumed to affect the pH mechanism specifically, also affect Cx-hemichannels and thus the ephaptic mechanism (Fahrenfort et al., 2009). As discussed in the next paragraphs, this is a highly complicated synapse involving many mechanisms working synergistically. To fully appreciate this synapse, all these aspects should be considered simultaneously.

### Two Feedback Pathways with Opposite Signs Work in Opposite Directions

In the dark, the Ca-current of the Panx1 mutants is activated at more hyperpolarized potential compared to WT. The results of this article demonstrate that mutating Cx55.5 or Panx1 have opposite effects on the activation potential of *I*_Ca_. This finding has significant consequences, since it shows that both feedback pathways work in opposite directions. In the dark HCs are relatively depolarized and the Panx1 channels are open. In this condition they release ATP which is converted into a phosphate buffer by NTPDase1 in the synaptic cleft and thus acidifies the synaptic cleft and inhibits the Ca-channels. Hyperpolarization of HCs leads to the closure of the Panx1 channels and ATP release stops and finally leads to disinhibition of the Ca-channels. This feedback pathway is thus maximally active when HCs are depolarized and in this maximally active state, it inhibits *I*_Ca_ and shifts its activation potential to positive potentials. This pathway behaves like a normal synaptic pathway being maximally active at depolarized potentials of the pre-synaptic cells: the HCs.

This is different for the ephaptic pathway. At depolarized potentials the driving force for the current through the hemichannels is smaller than at hyperpolarized potentials. Thus, the ephaptic feedback is maximally active at hyperpolarized potentials of the pre-synaptic cells. At hyperpolarized potentials, there is an increase in current through the hemichannels that leads to a stronger negativity in the synaptic cleft and to a shift of the activation potential of *I*_Ca_ to negative potentials, which is equivalent to a disinhibition of *I*_Ca_. This means that the ephaptic feedback mechanism is maximally active at hyperpolarized potentials and leads to disinhibition of *I*_Ca_. The Panx1 mechanism is maximally active at depolarized potentials and inhibits *I*_Ca_. Therefore, strictly speaking, the two feedback mechanisms, have an opposite sign and are inversely modulated by the HC membrane potential.

The question arises why you would have such an organization? This organization resembles the ON-OFF crossover inhibition pathways. The ON and OFF signals are sent to their downstream targets with opposite signs via separate cells and combined via synapses with opposite effects on their down-stream target. It has been proposed that such an organization is beneficial since it compensates for nonlinearities and reduces noise generated in each of the pathways (Molnar et al., 2009). This works best if the kinetic properties of the two pathways are rather similar. However, in the case of the two feedback pathways, the time constants of the two pathways differ considerably. This would mean that the above argument only holds for low frequencies. However, this is the first synapse in the retina and since the signal is coded in graded potentials, keeping noise levels low is of very high relevance.

### Time Constants of the Two Feedback Pathways

Vroman et al. (2014) showed that feedback from HCs to cones consists of two pathways with different time constants. The ephaptic feedback pathway has no synaptic delay while the other pathway has a time constant of about 300 ms in zebrafish. Interestingly this time constant of this slow process corresponds with the time constant of the pH change reported by Wang et al. (2014) using genetically encoded pH sensors in the synaptic cleft. The existence of two feedback pathways has been confirmed by Warren et al. (2016b) who also needed two time constants to fit the light induced feedback response in salamander cones adequately. The time constants they found for the slow feedback component was in the same order of magnitude as reported by Vroman et al. (2014). However, Warren et al. (2016b) estimated the time constant of the fast feedback component to be around 9–13 ms which is much slower than reported by Vroman et al. (2014), and suggests that the feedback synapse is rather slow. Why did these two studies report such different kinetics for the fast feedback component? Chapot et al. (2017) argued that the main difference between the two sets of experiments was that Warren et al. (2016b) induced feedback by polarizing a single HC whereas Vroman et al. (2014) did it by polarizing all HCs. This is not a trivial difference. Cones are contacted by a number of HCs and HCs are connected with each other by gap-junctions and often also indirectly via feedback pathways via the cones. The feedback signal measured in a cone is the sum of all the feedback signals from the various HCs received by that cone. That the feedback signal in cones is a mixture of inputs from various HCs was recently confirmed by Grassmeyer and Thoreson (2017) who showed that the various ribbons in a cone are affected differently when one modulates one HC. Polarizing only one HC leaves uncertain what the feedback signal from the other HCs is. Since the feedback signal generated by the (electrically or synaptically) coupled HCs will always be slower than that generated by the HC that was polarized, the estimate of the time constant of Warren et al. (2016b) will be an overestimate.

Vroman et al. (2014) used another approach. They used white light to hyperpolarize all HCs simultaneously. Thus a single cone would receive a similar feedback signal from all HCs that contact that cone. However, light responses of HCs have a time constant of about 35 ms. Therefore, estimating the time constant of the feedback pathway by just fitting an exponential function through the feedback response in the cone cannot be used. Instead, Vroman et al. (2014) applied another method for determining the kinetic properties of the feedback response. The amplitude/frequency and phase/frequency relations of the HCs, and the feedback signal the cones receive, relative to the stimulus, were determined. The amplitude data can be used to determine the filter characteristics of the synapse and the phase data can be used to determine the synaptic delay. In the article by Warren et al. (2016b) it is mistakenly mentioned that the slow kinetics of the HCs limit such an analysis. It is important to realize that a phase shift in the feedback signal is independent of how fast the HC response is. That is, if HC responses to a stimulus are slow this will be reflected in their phase shift data. Then if HCs relay their signal back to cones with an additional delay, the feedback response will show a larger phase shift relative to the stimulus than the HCs did. However, if the phase shift of the HC and feedback responses show the same phase shift relative to the stimulus it indicates no additional delay is added between the two. Based on the phase data, Vroman et al. (2014) showed that there was no synaptic delay and no additional filtering present in the feedback synapse from HCs to cones.

The mechanism of feedback has puzzled the retinal community for many years now. The picture emerging from this and previous studies is that HCs feed back to cones via two pathways: a fast ephaptic and a slow pH dependent mechanism. Whether both pathways are equally strong in the various animal models needs to be determined, but it seems reasonable to assume that animals will have adapted their feedback system to the visual environment they live in and the visual needs they have. Animals with relative low visual acuity, like mice, might not need a well developed fast ephaptic feedback system for spatial redundancy reduction because spatial vision is not well developed, whereas animals that strongly depend on vision, like zebrafish, might have a very well developed fast feedback system.

## Author Contributions

VC, WdG, TC, MP, GZ and MK: design of the experiments, conducting the experiments, analysis of the results and writing the article.

## Conflict of Interest Statement

The authors declare that the research was conducted in the absence of any commercial or financial relationships that could be construed as a potential conflict of interest.

## Footnotes

^1^www.ectonucleotidases-ab.com
